# Exploring Cancer Patients’ Experiences of an Online Mindfulness-Based Program: A Qualitative Investigation

**DOI:** 10.1007/s12671-020-01380-z

**Published:** 2020-04-27

**Authors:** Brittany A. Glynn, Eve-Ling Khoo, Hayley M. L. MacLeay, An Duong, Rosemee Cantave, Patricia A. Poulin

**Affiliations:** The Ottawa Hospital Research Institute, The Ottawa Hospital – General, Smyth Road, Ottawa, ON K1H 8L6 Canada

**Keywords:** Chronic pain, Mindfulness, Qualitative research, Online MBP

## Abstract

**Objective:**

Chronic neuropathic pain (CNP) is a common condition cancer survivors experience. Mindfulness training may be one approach to address the psychosocial factors associated with CNP. The purpose of this study was to understand patients’ experiences in an 8-week online mindfulness-based program (MBP), including techniques and skills learned and applied, barriers to practice, and research experiences.

**Methods:**

Nineteen participants who were part of a randomized controlled trial consented to participate in a telephone interview or submit written responses via email post-course. Interviews were transcribed and analyzed using the principles of Applied Thematic Analysis (ATA).

**Results:**

Predominant themes identified in participant interviews included (1) common humanity, (2) convenience, (3) teacher resonance, (4) perceived relaxation and calm, (5) pain and stress management, (6) half-day session, and (7) mindful breathing. Participants also identified helpful strategies learned and implemented from the course, as well as barriers to practice, and key components of their experiences in a randomized controlled trial, including a sense of disconnection post-course and needing continued ongoing sessions, and the importance of the facilitators’ skills in creating a comfortable and supportive space.

**Conclusions:**

An online group-based MBP may offer a more accessible resource and form of psychosocial intervention and support for cancer survivors living with CNP. Furthermore, the need and consideration for implementing ongoing group maintenance sessions to minimize participants’ feelings of disconnect and abandonment post-course and post-study are warranted in future MBP development.

Chronic neuropathic pain (CNP) is common among cancer survivors (Ngamkham et al. 2019). This is due to nerve damage that may be caused by cancer treatments, including surgery, radiation, chemotherapy, or tumor infiltration. Postsurgical neuropathic pain syndromes affect between 20% and 50% of cancer survivors (Rogers and Duffy 2000; Stevens et al. 1995), while toxicities from chemotherapy agents can lead to acute chemotherapy-induced peripheral neuropathy (CIPN) (Quasthoff and Hartung 2002). A recent review indicates that CIPN affects more than 60% of patients, with 30% of patients reporting symptoms lasting more than 6 months after treatment completion (Seretny et al. 2014). Additionally, CNP is often associated with depression, anxiety, and insomnia (Jensen et al. 2007) and the severity of CNP correlates directly with disability and reduced health-related quality of life (Burckhardt and Jones 2005; Jensen et al. 2007).

CNP is complex to treat and like many other chronically painful conditions, an interdisciplinary or holistic approach addressing both biological and psychosocial factors that may contribute to the overall pain experience is indicated (Scascighini et al. 2008). Mindfulness training is one approach that may be helpful for cancer survivors living with CNP.

Mindfulness can be defined as a “non-elaborative, non-judgmental, present-centered awareness in which each thought, feeling, or sensation that arises in the attentional field is acknowledged and accepted as it is” (Bishop et al. 2004). While there is considerable research demonstrating benefits of mindfulness-based programs (MBPs) in chronic pain, there is comparatively little work that has been done for cancer-related pain (Khoo et al. 2019). Ngamkham et al. (2019) found that there are potential benefits for pain and psychological distress (i.e., anxiety, depression, and stress). Additionally, two recent meta-analyses of MBPs for cancer patients and survivors have both reported improvements in pain, psychological distress, anxiety, depression, fear of cancer recurrence, and sleep quality (Cillessen et al. 2019; Zhang et al. 2019). Of note, four of the 29 studies included were online MBPs where no significant changes in the pooled results were observed after a sensitivity analysis (Cillessen et al. 2019). However, Cillessen et al. (2019) also noted that there is a need for more intervention optimization research given the variance in cancer populations and protocol adherence to different MBPs included in the meta-analysis.

More recently, there has been an interest delivering online video conference MBPs for cancer and pain. Traveling to in-person classes is a burden on those dealing with cancer (Kinner et al. 2018; Smith et al. 2005). When compared with an in-person mindfulness-based intervention, online MBPs are frequently determined to be similarly effective (Gardner-Nix et al. 2008; Garrido-Torres et al. 2016). Both cancer and pain populations report improvements in anxiety, depression, and pain intensity (Cillessen et al. 2019; Dowd et al. 2015; Messer et al. 2019), where cancer patients report additional improvements in fear of cancer recurrence, sleep, and fatigue (Cillessen et al. 2019) and chronic pain patients reporting decreases in total pain interference, pain catastrophizing, and increases in pain acceptance (Dowd et al. 2015; Hearn and Finlay 2018). Both cancer and pain literatures report ongoing benefits following online treatment completion (Cillessen et al. 2018; Dowd et al. 2015).

The call for better understanding the underlying mechanisms and processes of mindfulness practices has warranted the use of qualitative methodologies to explore cancer patient experiences and the effects of MBPs (Shennan et al. 2011; Zimmermann et al. 2018). Studies attempting to elucidate this have largely focused on women with breast cancer (Schellekens et al. 2016; Tate et al. 2018), with some exceptions (Kinner et al. 2018; Mackenzie et al. 2007). The majority of interventions followed the standard 8-week mindfulness-based stress reduction (MBSR) program (Kabat-Zinn 1990, 2003; Tate et al. 2018). However, several studies have used modified MBPs incorporating elements of mindfulness-based cognitive therapy (MBCT) (Chambers et al. 2012) and acceptance commitment therapy (ACT) (Kinner et al. 2018). Most mindfulness studies examining the processes and mechanisms of mindfulness using qualitative approaches have been done with in-person mindfulness training, though some explored online interventions where participants and the facilitators join from home or remote locations (Kinner et al. 2018) or via telemedicine groups (Gardner-Nix et al. 2008). As such, there are currently no studies investigating the experiences of an online MBP for CNP in cancer patients.

Of the mindfulness studies exploring cancer or chronic pain participants’ experiences in either in-person and online interventions, several common themes arose. In both online and in-person interventions, the group aspect proved beneficial to the majority of participants, who reported feeling connected, understood, and safe to express themselves (Kinner et al. 2018; Penlington 2019; Tate et al. 2018). Additional themes in the qualitative literature were the acquisition of new emotion regulation and psychological skills such as increased acceptance (Penlington 2019; Tate et al. 2018), presence (Tate et al. 2018; Weitz et al. 2012), awareness (Luiggi-Hernandez et al. 2018; Tate et al. 2018), self-care and self-compassion (Penlington 2019; Tate et al. 2018), self-control, and decreased reactivity (Penlington 2019; Tate et al. 2018), as well as stress-management and related coping skills (Penlington 2019; Tate et al. 2018). The development of these skills has been associated with re-perceiving (Luiggi-Hernandez et al. 2018; Tate et al. 2018), posttraumatic growth (Mackenzie et al. 2007; Weitz et al. 2012), decreased depression and anxiety symptoms (Kinner et al. 2018; Penlington 2019), an improved relationships with self (Penlington 2019; Tate et al. 2018), and increased quality of life (Kinner et al. 2018; Tate et al. 2018). Participants have also developed skills as a result of participating in mindfulness training, as well as maintained mindfulness practices post-course, including breathing techniques (Penlington 2019; Tate et al. 2018), body scan meditations (Luiggi-Hernandez et al. 2018), mindful eating (Eyles et al. 2015), and overall improvements in emotional self-regulation skills (Penlington 2019; Tate et al. 2018).

Conversely, challenges have been identified for both in-person and internet-delivered MBPs across different populations. These include the time commitment required (Kinner et al. 2018; Tate et al. 2018) and the physical limitations of participating in mindful movements (Tate et al. 2018). Technological issues can be a challenge for Internet-delivered mindfulness treatments (Kinner et al. 2018). Participants also find it challenging to continue to practice mindfulness in their everyday lives following the end of the course (Berk et al. 2018).

Additionally, there is growing interest in understanding potential adverse effects and negative health outcomes participants may experience from engaging in MBPs, including difficulty managing intense feelings and troubling thoughts, and increased perceptions of negative experiences of self (Lomas et al. 2015; Tate et al. 2018). Although rates of serious adverse events are thought to be low and may parallel the rate of occurrences seen in general psychotherapy (Crawford et al. 2016), the occurrences nonetheless warrant further attention to the special characteristics of individuals and vulnerable populations participating in MBPs who may be more or less susceptible to the negative and positive effects of the mindfulness and meditation practices. In regard to chronic pain populations, patient vulnerabilities and beliefs about their capabilities to control or diminish their pain through mindfulness and meditation are also worth consideration for creating potential negative effects, particularly in developing false beliefs and expectations of the practice outcomes. Furthermore, the potential for negative experiences may be a repercussion of inadequate understanding and teaching practices from instructors, thus warranting a need for future research to explore participant experiences and the conditions that create greater risk for adverse effects with careful consideration of the participant population (Van Gordon et al. 2017).

This study aimed to explore the experience of cancer survivors living with CNP who participated in an online MBP, including techniques and skills learned and applied, barriers to practice, and participant experiences in a long-term psychosocial intervention research study. Research questions guiding this study included what is the experience of an 8-week online MBP for cancer patients who experience CNP, what strategies and techniques do participants learn and apply from an online MBP in relation to pain management, what barriers impact practicing mindfulness meditations during the program and maintaining a practice post-course, and what is it like to be in a randomized controlled trial (RCT) from the participants’ perspective and how can more support be offered to participants?

## Method

### Participants

Participants were a part of a RCT investigating an online 8-week MBP for cancer survivors at any stage of the disease trajectory and who had CNP. The CNP had to be related to their cancer or cancer treatment, present for at least 3 months, and rated as moderate to severe (score ≥ 4) based on either the pain intensity or pain interference scale of the Brief Pain Inventory. The diagnosis was confirmed by a pain physician prior to entering the study. The participants had to be able to attend seven out of the nine MBP course sessions (e.g., 8 weekly 2-h session and 1 half-day session) and complete the course and questionnaires in either English or French. In addition, due to the nature of the online course, participants must have had access to the Internet and use of an electronic device capable of joining a video conference. Exclusion criteria included a prognosis of less than 12 months, cognitive impairment, severe psychiatric disorder impacting ability to participate (e.g., schizophrenia, severe depression), or prior experience participating in a MBP.

Of the 24 participants who were contacted from the larger study after their post-MBP questionnaires were completed, 19 consented. Recruitment stopped once eighteen participants completed the telephone interviews and one participant submitted written feedback via email due to being unable to participate in the phone interview. Participants ranged from 31 to 68 years of age (mean = 55.2) where the majority were women (72.2%), had post-secondary education or greater (77.8%), were married or in a common-law relationship (61.1%), and were not presently working (i.e., retired, unemployed, and/or on disability leave or a disability support program) (83.3%). All the participants were Caucasian except for two who identified as other ethnicities. On average, participants reported living with pain for 1.8 years (SD = 2.3) and ranging from 5 months to 10 years. Half of the participants reported breast cancer as their primary cancer diagnosis. See Table 1 for more demographic information.

**Table 1:** Participant demographics

Variable, *n* (%)	Total *n* = 18 (%)
Age, mean (SD), y	55.2 (SD= 11.2), range 31-68
Female	13 (72.2)
Ethnicity	
Caucasian	16 (88.9)
Other	2 (5.6)
Education	
≤ High School	4 (22.2)
Post-secondary/Technical diploma	7 (38.9)
Bachelor’s degree	5 (27.8)
Master’s degree	1 (5.6)
Doctoral degree	1 (5.6)
Employment	
Working^a^	3 (16.7)
Unemployed	3 (16.7)
Unemployed with ODSP	1 (5.6)
Retired	6 (33.3)
Retired with ODSP	1 (5.6)
Retired and on disability leave	2 (11.1)
Disability leave	1 (5.6)
Disability leave with ODSP	1 (5.6)
Marital Status	
Single	4 (22.2)
Married/Common-law	11 (61.1)
Divorced/Separated	3 (16.7)
Pain Duration, mean (SD), y	1.8 (2.3), range = 0.4-10
Primary Cancer Diagnosis	
Breast cancer	9 (50)
Colorectal cancer	2 (11.1)
Testicular cancer	2 (11.1)
Other^b^	5 (27.8)

^a^Employment subcategory *Working* indicates full-time (1) and part-time (2)

^b^Primary cancer diagnosis subcategory of *Other* indicates adenocarcinoma (1), cervical cancer (1), colon cancer (1), lung cancer (1), and rectal cancer (1)

### Procedure

Prior to the start of the 8-week MBP course, participants met with research assistants to download and receive a tutorial for using the videoconference platform (e.g., Zoom Communications) and platform features, such as turning the sound and online video on or off. The participants and their teacher then met via videoconferencing for 2-h once a week for 8 weeks straight. The half-day session was also done online and held halfway through the course. The duration of the half-day session was modified to 3-h given the online nature. The course content was taught by a certified mindfulness teacher and included in-class meditations, group inquiry, didactic discussions (e.g., body, emotions, sensations, thoughts, and compassion in relation to pain), as well as various mindfulness activities and meditations that participants were encouraged to practice daily as part of their home practice. Two facilitators were represented in this study. Following the end of the 8-week course, participants who had completed their post-MBP questionnaires (at least 2 weeks post-MBP) were contacted by email by a research team member asking for their consent to participate in a telephone interview. Once consent was established, the telephone interview was performed.

### Measures

One research team member who is also one of the coauthors (EK) completed the interviews. All telephone interviews were recorded with the participant’s consent and lasted from 6 to 75-min with the exception of one participant who emailed typed responses to the interview questions. The semi-structured interviews consisted of open-ended questions pertaining to the participant’s experience in the online MBP, as well as in the research study. See Table 2 for a list of the interview questions. The interviewer could probe for further clarification and explanation when necessary. Interviews were then transcribed and checked by two members of the research team. The research team member performed 18 telephone interviews and obtained data saturation (Guest et al. 2006).

**Table 2:** Telephone interview question

Please tell me about your experience in the 8-week mindfulness course. Was there anything particularly memorable for you?	
Were there strategies that you learned that have been helpful for your pain management or quality of life? If so, could you describe them and give examples of how you are using them?	
Have you been practicing any of the mindfulness techniques since your class ended (e.g., awareness of your breath/body; meditations, mindful walking; or mindfulness of a daily activity, etc.)?	
Is there anything else you would like to share about your experience in the mindfulness course?	
Do you have any recommendation for how to improve participant experience in future studies?	
Did you receive enough support from the research staff regarding the technological platform, research appointments, and online questionnaires?	
Is there anything else you’d like to share about your experience in this study?	

### Data Analyses

All interviews were de-identified, transcribed, checked by a research team member (BAG) for accuracy, and uploaded into QSR NVivo10 qualitative data analysis software (NVivo qualitative data analysis software 2012). The principles of Applied Thematic Analysis (ATA; Guest et al. 2012) using a thorough step-by-step analysis were employed, including (1) identifying significant text via reading and re-reading the interview transcripts, (2) considering emergent meanings, (3) identifying and labeling potential themes, (4) coding the text line by line, (5) comparing and contrasting themes among transcripts, and (6) reviewing and developing thematic patterns. Four members of the research team independently read and coded all of the transcripts and each member developed preliminary themes. The codes and development of the overall thematic scheme were then discussed among the four research team members at weekly coding meetings.

## Results

Analysis of the interviews focused on four areas: (1) participant experiences of the 8-week online MBP course, (2) strategies and what participants learned from the course, (3) barriers to practice and ongoing practice, and (4) participant experiences in the research study. Thematic schemes and sub-themes in each of these are as follows.

### Participant Experiences of the Online Course

Seven predominant themes emerged: (1) common humanity, (2) convenience, (3) teacher resonance, (4) perceived relaxation and calm, (5) pain and stress management, (6) half-day session, and (7) mindful breathing (Table 3). Thirteen participants noted the common humanity experienced in the group setting as a predominant positive aspect of their experience in the course. They reported feeling a sense of connection to the other participants and feeling less isolated being in a setting with other people who were also experiencing CNP. As one participant explained, “I was glad for their presence, that’s what I was glad for…and that they were listening […] it was good to be with people that um- you know had pain also.”

**Table 3:** Experience in the course

a. Common Humanity • Participating in this study in a group who also have chronic pain, made me realize, that I’m not the only one going through that, but there are others like me. – Participant J • Everybody was just sort of on the same level. And um, it was very good for me to see, and to participate with other people that were also having pain because it made me realize that I wasn’t alone. – Participant O • ...um speaking with other people is something I enjoy doing cause you do not necessarily get to talk to other people a lot all the time about the cancer they are going through that have similar experience to you, especially that type of environment. So, I really liked being able to see people and hear them, yeah being together, and seeing everybody else’s experience. – Participant A	
b. Convenient • I really enjoyed it. The uh, online experience was new for me. I had not done conference calls before. And uh, I enjoyed how easy it was to just sign up and like sign on and start talking with people ...you know how I do not have to exert myself or make like too much of an extra effort to go out, especially when I’m not feeling well. – Participant D • I also enjoyed being able to do it from home at that time of year, was an awesome accommodation for my physical status. – Participant E • It was really nice to have to do it on Zoom. For the most part we could decide whether to show ourselves. – Participant A • It’s so much easier to you know seven o’clock or six o’clock you turn on your computer, you are there, it’s all good. – Participant R • For me I adored the Zoom technology platform. We were meeting and sharing experiences without commuting. – Participant J	
c. Teacher Resonance • ...the instructor did a very good job. She was patient in listening to what each participant had to say even though they rambled on a bit. She was good at taking the information and bringing it back to the subject matter. – Participant L • I enjoyed the instructor a lot. He was excellent. He had this knack of being able to take something that was brand new to me and I believe to the other participant in my class as well, from what I could understand. And so he could take a concept that was really new, explain it really well and simply, yet also challenge. – Participant E	
d. Perceived Relaxation and Calm • I found it very relaxing. I found that it was nice way to learn to relax and unwind when I’m getting tense with situations. – Participant F • In the beginning I found it hard to relax, and throughput the course I was able just to let go, and focus, and just taking time out for myself and being able to relax. I was having problems sleeping at night and relaxing during the day and throughout the course, the mindfulness course, I was able to accomplish that. – Participant B	
e. Pain and Stress Management • ...every once in a while, I’ll uh, you know, take a few moments for some awareness of my situation or my surrounding, or you know, do a quick little analysis like okay... recognize that there’s a little bit of pain in my lower back. It exists, it’s welcome it exists, but you know, go to somewhere else and be like well this is a place that does not have pain. I find actually it helps me calm down a little bit. – Participant D • Yeah, every time I hear that it’s just 'Just breathe.’ (*Laugh*). And it’s really helped me through the whole cancer scenario because every time I start to feel anxious about something that was happening, I would just tell myself that – to repeat those words. – Participant F • I mean I wasn’t sure what to expect going into it but it certainly touched on a lot of factors that were important in helping me cope with everything that was going on. And um you know I did not feel um like I was thrown into a whirlwind. It helped me kind of feel like I had, you know; could get my head above water and um manage everything that I was coping with. – Participant I	
f. Half-Day Session • ...because it was a lot more intensive. It allowed me to get into kinda a deeper mindset, and I was really able to- I guess I wasn’t always able to do the body scan, but on *that* day, I could get fully into the body scan., And yeah, the whole day, and I’d say even the whole weekend after that was very uh- just calming and serene... – Participant D • I really liked the half- the uh- workshop that was included... we did sort of like a half-day session, and that was really valuable to get sort of an extensive piece. – Participant E • ...um only because it’s just another um you know, the meditation for three hours was a little bit too much for me, And at first, after about an hour, I’ve had about enough, and I thought, “Okay well maybe let us go through this and see what happens”, and after the three hours, I was actually quite happy that I had gone through the three hours. I found that it was...how do I say this...beneficial to me even though I did not think I would find it beneficial. – Participant L	

Nine participants noted convenience as an integral aspect of their experience in the online 8-week course. Convenience included participants appreciating the online format and being able to participate in the class from the comfort of their own home or another chosen location. As noted by one participant “…I enjoyed how easy it was to just sign up and like sign on and start talking with people…I don’t have to exert myself or make like too much of an extra effort to go out, especially when I’m not feeling well.” Participants also had the option of turning their camera off for privacy or leaving it on and showing their faces. Additionally, participants appreciated that the class included activities and practices that were easy to implement, such as the 3-minute breathing space meditation or breath-focused techniques, which could be used “in the moment” when experiencing pain or distress.

Eight participants noted the teacher as part of their experience in the course and seven participants specified the teachers’ facilitation styles. Participants appreciated the teachers’ abilities to integrate and connect the participants’ experiences with the course content and take abstract concepts and explain them in understandable ways, yet also challenge the participants to further reflect on their own experiences. Furthermore, teacher attributes, such as attentive listening and a calm and present demeanor, were meaningful to the participants.

Perceived relaxation and feeling calm included noticing a reduction in tension and a sense of “letting go” of stress and difficult situations. Relaxation was noted physically, such as less tension in the body, as well as emotionally and feeling less distress when experiencing frustration or sadness related to pain. Additionally, some participants noted improved sleep quality and falling asleep more easily.

Pain and stress management included the participants finding ways to cope and be with their pain in more skillful ways. For example, one participant noted feeling lower back pain and then intentionally bringing attention to their surroundings and other areas of the body that were not in pain. Some participants noted a decrease in their neuropathic pain, and as one participant stated: “A lot of my neuropathy pain has subsided as well, I would say about 80% right now.” Other participants explained feeling less anxious and overwhelmed during difficult situations and feeling better able to cope with worries and stressors related to their cancer diagnosis, as well as stressors related to other areas of their lives.

The half-day session was also a predominant theme. Participants explained that the longer duration of practice allowed them to go “deeper” with the meditations and increased perceptions of feeling calm and serene. Some participants also noted their initial skepticism and apprehension of engaging in an extensive day of practice, but then found the day to be beneficial and appreciated their ability to participate in prolonged practice.

### Helpful Strategies Learned and Implemented

Several strategies were considered helpful and implemented by the participants. Strategies learned included the following: developing skills in awareness; mindful practices, such as mindful breathing, mindful eating, and mindfulness of daily activity; and intentionally pausing and slowing down (Table 4).

**Table 4:** Helpful strategies learned and implemented

a. Awareness • After a while I decided to stop, take a break, and instead respond in a skillful way. I put down all thing to practice mindful consumption, the awareness on the things I watch, listen and read. I told myself “let me change all the strategies.” I started watching fun shows, stand-up comedy, listen to music, and a lot of fun things. Once I was in awareness and not in [autopilot], the anxiety, stress and anger started to dissipate slowly and got replaced by curiosity pleasure and even joy. – Participant J • I’ll take a few moments for some awareness of my situation or my surroundings, or you know, do a quick little analysis like okay... recognize that there’s a little bit of pain in my lower back. It exists, it’s welcome, it exists, but you know, go to somewhere else and be like well this is a place that does not have pain, I find actually it helps me calm down a little bit. – Participant D • I came to the realization of the fact that being out in nature was-uh like meditating out in nature, meditative walks, um and just the fresh air. I guess I just underestimated the value of that in my life. And so I was able to use that um more and more- that had an incredible effect on me and my wellbeing... I guess I just underestimated (*laughs*) how much that can help me, you know, both mind and body. – Participant I	
b. Mindful Practices • Mindful Breathing - I will say the um the breathing-the visualization. I actually try and every morning, um you know, take the time to relax, try and be connected with the outdoors in some way. And it just comes down to uh slowing things down um the breathing, using the breathing techniques. – Participant D – What I will do is, if I found myself rushing during the day, then if I think about it I will stop and then I will take a few breaths. – Participant L – Well especially the breathing because some of the procedures I go through is quite painful, so I can just turn myself off and do the breathing and focus on me – what’s going on around me. I find that really helpful if you could just turn yourself off and learn to relax at a different pace. – Participant B • Mindful Eating – I’m more mindful about what I eat, which is interesting because I give more thought to what goes into my body, as opposed to before where it was just well I’ll eat whenever you are hungry, or I’ll eat whatever happens to be around. – Participant L – I’m just a lot more mindful about what I put in my body. – Participant A • Mindfulness of a Daily Activity – These techniques have helped me find peace and stay out of depression and anxiety. I also learned to develop the capacity to focus on the moment, whether I’m walking, exercising, or eating, without having my mind to wander. – Participant J – I do the breathing every morning and when I’m walking. I take the dogs out for walks and sort of enjoy the sunshine, or like instead of mourning about the cold (*both laugh*) to sort of try and see the positive in that. – Participant C	
c. Pausing and Slowing Down • I guess stopping and, you know, just stopping and listening, stopping and looking. – Participant I • I think the idea of sort of slowing down, taking a breath. – Participant G • I actually try and every morning take the time to relax, try and be connected with the outdoors in some way, And uh, it just comes down to slowing things down, the breathing, using the breathing techniques. – Participant I	

Awareness themes included body awareness, awareness of the environment, and awareness of daily activities. Participants learned how to become more aware of their body sensations, body positions, and postures. They also developed an increased awareness of their external surroundings, such as becoming more aware of their natural surroundings during walks or focusing on external stimuli during painful moments. They also developed awareness of the physical limits of their bodies, as well as mental and emotional limits. Subsequently, they then practiced respecting those limits and practicing self-care habits (e.g., pacing physical activities and resting when needed). As explained by one participant who was feeling consumed by fear of cancer reoccurrence and constantly watching online videos and documentaries, “After a while I decided to stop, take a break, and instead respond in a skillful way. I put down all things to practice mindful consumption, the awareness on the things I watch, listen and read” which helped her respect her emotional boundaries, rather than over-consuming information that contributed to her fear of reoccurrence.

Mindful practices included mindful breathing, mindful eating, and mindfulness of a daily activity. Seven participants noted mindful breathing as a helpful strategy. Participants found focusing on their breath, particularly during difficult moments, to be helpful for “slowing down” and anchoring their awareness in the present moment. As explained by one participant: “Well especially the breathing because some of the procedures I go through [are] quite painful, so I can just turn myself off and do the breathing and focus on me- what’s going on around me…” Focusing on their breathing was the predominant coping strategy learned by the participants and used during experiences of pain and stressful situations.

Participants also noted greater awareness of what they were eating and consuming. One participant explained: “I’m more mindful about what I eat, which is interesting because I give more thought to what goes into my body, as opposed to before where it was just well I’ll eat whenever you’re hungry, or I’ll eat whatever happens to be around.” Participants became more aware of their consumption habits and the impact of food and other substances on their bodies. Additionally, participants became more aware of their daily activities, particularly in their physical activities such as walking or exercising.

Learning to pause and slow down was a predominant strategy. Participants referred to slowing down as a means of becoming more aware of their environment, actions, thoughts, and emotions rather than engaging in autopilot and automatic ways of thinking, feeling, and acting. They also used these moments to take an intentional breath and thus integrated mindful breathing.

### Barriers to Practice and Ongoing Practice

Six participants noted time as their greatest barrier to practicing during and post-course (Table 5). Making time to practice was considered challenging, particularly due to the participants’ competing priorities and obligations. As explained by one participant, “I think my life is extremely, extremely busy. And uh- and that is a barrier because I am uh- I am a caretaker to my husband.” Additionally, it was difficult to find the time to make practicing a regular habit (e.g., engaging in a regular formal or informal practice). Furthermore, external factors, such as cold weather and icy conditions, made it difficult for some to practice intentional awareness while walking outdoors in conjunction with experiencing neuropathy in their lower extremities.

**Table 5:** Barriers to practice and ongoing practice

a. Time • It’s just the hectic fast pace life that I live. – Participant R • Actually a lot of the time it’s cause I do not think of doing them. Like she would give us homework to do and we have to do this, and I sometimes would forget – cause you are so busy with things you just do not think about it. – Participant Q • I think- I think my life is extremely, extremely busy. And uh- and that is a barrier because I am uh- I am a caretaker to my husband. And uh- and I’m also um- I also participate in the lives of my two grandchild, two and five, so I’m very very busy with them. – Participant Q	
b. Habits • I guess the only barrier would be just it’s a challenge incorporating new habits. – Participant M	
c. External Factors • Well it’s kind of hard with the mindful walking because I’ve got peripheral neuropathy in my legs from the chemotherapy and the radiation that I had, so it’s impossible for me to walk in snowy conditions and icy conditions. So I... and there’s something else. A lot of the things we are asked to practice are weather dependent. So I had to think of substitutes. – Participant H	

### Experiences in a RCT of an Online MBP

All 19 participants noted that they felt supported during their participation in the research study, including support from the research team for study logistics, as well as support from their teachers during the course. However, participants did note several challenges and suggestions for improving participant experiences in the future. Five predominant themes emerged in consideration of the participants’ research experiences in the RCT, including (1) challenging questionnaires, (2) technological issues, (3) communication, (4) disconnection, and (5) continuity (Table 6).

**Table 6:** Experiences in the research study

a. Challenging Questionnaires • I found the questionnaires difficult because they asked the same thing but in sixteen different ways. So some of them is like ‘So what’s your interpretation of *that *word” or “that *level*.” I’m answering them and I’m kind of saying “Okay you know what, stop overthinking this, do what your gut feels and go. Cause it was a struggle each time to sort of figure out ‘Okay, what do they mean by that?’ ‘Oh okay (*both laugh*) ... I get it.’ – Participant F • I think that overall the entire experience was very good. I found that if was uh, for the questionnaires, I found it difficult in that a lot of the pain that I’m experiencing is not as a result of the cancer. It is a result of osteoarthritis. So a lot of times I did not know because the questionnaire does not specifically say, “In your case with the treatment for this.” You do not know if you are answering with regards to your overall pain, or if you are answering with the pain for example the neuropathy, which was caused by the chemo. So I found that difficult and I wished there was a clear line in that you were answering for specifies in that way. – Participant L • The questionnaire... they seem long and repetitive. – Participant M	
b. Technological Issues-Sound • ...our instructor’s microphone sometimes had a lot of static and distortion so it made it harder to hear them. So maybe like a better quality microphone might have helped. – Participant D • There were technical difficulties and the main difficulty was that some people, including myself, but some people even more so, could not hear our instructor. The sound was fine and when I first started, as I said, the sound was a problem. I had it right up to the top and [the teacher] was very muffled...and I believe it was because of the microphone but that was adjusted... – Participant 0	
c. Communication • ...I really had absolutely no idea what the course was about...so if there was a little more introduction to the course... – Participant 019 • I really did not know what to expect. I think we were told it was [teacher] just before but there was no, “[Teacher] has had experience in dah dah and she works for the university”, and yes, we didn't know. I don't believe we knew who we were getting until the lasty minute. I think that would be helpful cause I think people would like to know... I think that was a concern of mine. – Participant 0	
d. Disconnection • I think that maybe a lot of people, including myself, are sort of left hanging. And we are saying “Well this has been wonderful, we are done this eight-weeks, or however long it was, and now it’s finished what now?” You know? - Participant O • But I felt abandoned. So the end of the eight weeks,. there was a sense of abandonment because I felt that the course really served as a pre-course to something else. There had to be something else. There has to be a stage two to this. So I just got to the point where I was reconsidering lifestyle, I was reconsidering who I was, I was thinking about things in a different way and attempting to do things differently, and then it all ended. There didn't appear to be much follow up. – Participant H	
e. Continuity • It would be kind of fun to pull people back, you know after a year and do a little refresher. I would think- cause probably in my little rut you know, doing stuff and I’ve forgotten stuff. And a little refresher I think, just talking for my two cents, would be really beneficial. - Participant E • I found it a very positive experience, and I know that there are resource limitations, but I thought that it ended prematurely. – Participant H • ...we had the weekly session and then we went from that to pretty much nothing. I think it would’ve been nice[...] once that was over, if we could have a session maybe once every two weeks, and then maybe once every three weeks, and you know, maybe not cut it off *completely*. So that we can kind of regroup and stay connected and have that schedule time maybe, you know, once a month so that it’s more at the forefront. –Participant I • ...if you can have a session maybe every six months, or something, some little refresher thing because I think that would help, it might actually engage people more. –Participant P	

Seven participants found the questionnaires sent to participants at multiple assessment points challenging. Issues included the questionnaires taking a long time to complete and asking redundant questions. Furthermore, participants were uncertain at times if they were answering about their pain levels in general or specifically their neuropathic pain despite instructions, as many of the participants had co-existing pain problems related to other conditions or generalized pain in addition to their neuropathic pain symptoms. As noted by one participant: “You don’t know if you’re answering with regards to your overall pain, or if you’re answering with the pain for example the neuropathy, which was caused by the chemo. So I found that difficult and I wished there was a clear line in that you were answering for specifics in that way.”

Eight participants found aspects of the online technology challenging, specifically related to the Internet connection being interrupted from their computer or the teachers’ and the sound from the teachers’ microphones being muffled or non-existent at times and they could not hear what the teacher was saying. Participants suggested that the research team should explore better ways to support the audio component of the course.

Seven participants noted communication throughout the study to be important to them and at times lacking, specifically in providing more information and specific details about the course and their teacher. Furthermore, although participants were informed during the consent process about not receiving their individual test results from the study, three participants explained that they would have liked to see and receive explanation about the meaning of their test results.

Finally, although participants were given several resources to continue to practice mindfulness, three participants noted feeling disconnected and a loss of direction once the courses finished, specifically stating that they were “left hanging” and “abandoned” (see Table 6). Participants would have liked more follow-up after the class finished, whether this was staying connected to the other class members or community groups. Likewise, continuity and having refresher courses or meetings post-course were important to the participants. The 8 weeks were not long enough for some of the participants. As one person explained, “…we had the weekly session and then we went from that to pretty much nothing. I think it would’ve been nice […] once that was over, if we could have a session maybe once every two weeks, and then maybe once every three weeks, and you know, maybe not cut it off *completely*.”

## Discussion

This study is the first to investigate the experiences of an online MBP intervention from the perspective of CNP cancer patients and contributes to a better understanding of patient experiences. As part of a larger RCT, this study brings attention to the most salient components of participating in an online 8-week course, as well as what strategies they learned and what barriers participants experience in their efforts to practicing mindfulness and maintaining an ongoing practice. Additionally, this study contributes to our understanding of conducting RCTs with cancer patients and their experiences of participating in longitudinal research studies focusing on psychosocial interventions. The findings from this study may help inform future MBP facilitators and researchers when working with chronic pain and cancer patient populations.

The group setting and feeling common humanity were critical aspects of the participants’ experiences. Participants felt less isolated and alone when listening to their classmates share about their experiences, in addition to being able to share about their own experiences. This coincides with other studies showing the group aspect to be very important, particularly because participants felt understood, connected, and able to express themselves in ways that they may not be able to in other aspects of their lives (Kinner et al. 2018; Tate et al. 2018). Aside from learning mindfulness skills and techniques, online courses may help participants feel more socially supported with their pain and cancer experiences. An important aspect of the participants’ experiences was the convenience of the course (e.g., online delivery) and convenience of the practices and meditations. There was a sense of comfort in being able to participate in the class from the comfort of their home, while also being able to choose if they wanted to show their face by turning off their camera for privacy. The online delivery format may offer a more accessible resource for CNP patients, as previous research demonstrates that physical limitations, including a lack of mobility and decreases in energy, may make in-person classes more challenging for cancer populations (Kinner et al. 2018). Additionally, the privacy option was helpful so participants could attend and engage with the practices, yet also honor how they were feeling and take care of themselves while undergoing cancer treatments (e.g., chemotherapy and radiation). Furthermore, participants found several mindfulness meditations and techniques to be useful “in-the-moment,” such as the 3-minute breathing space or breath-focused practices. Practitioners developing MBPs, courses, and tools for CNP patients may consider the convenience of the course delivery format and what activities and tools may be most accessible and convenient for this specific population’s needs.

This study also highlights the role of the teacher and facilitation styles for online 8-week courses. In addition to the perceived benefit from being in a group setting, the teachers’ calm and attentive demeanors were particularly helpful to the participants, as well as the ways the teachers could integrate their shared experiences within the course curriculum and discussion topics. Previous research demonstrates that mindfulness teachers’ experience of mindfulness, personal practice, and presence are key elements of program success and mindfulness pedagogy (Crane 2009; Crane et al. 2010; Kabat-Zinn 1990, 2003, 2005; Kabat-Zinn and Santorelli 2005; McCowen and Reibel 2009; Segal et al. 2002).

Perceptions of relaxation and calm, as well as developing ways to manage pain and stress, were predominant aspects of the participants’ experiences. Other studies have demonstrated MBSR course participants learning new psychological skills that have reduced feelings of depression and anxiety, improved sleep and quality of life, as well as finding better ways to cope with illness and everyday stressors (Kinner et al. 2018; Luiggi-Hernandez et al. 2018; Penlington 2019; Tate et al. 2018). Although the focus was on CNP, these online courses may offer participants skillsets that are applicable to multiple areas of their lives beyond pain management as healthy coping skills.

The half-day session was another key aspect of the participants’ experiences. Similar to previous studies (Tate et al. 2018), participants in this study noted the length of the half-day retreat being long, however, they also reported significant benefits from the half-day session, such as appreciating the opportunity to go deeper with the practices. Of consideration is that the walking meditation portion was to be completed on the participant’s own time, thus the duration of the online retreat was shorter than the standard half-day length for in-person classes. Condensing the half-day retreat so that it is suitable for chronic pain patients is worthy of consideration for the needs of this population and creating more opportunities for the participants to take breaks and take care of their physical needs.

Similar to other studies (Luiggi-Hernandez et al. 2018; Penlington 2019; Tate et al. 2018; Weitz et al. 2012), mindful breathing was the most common strategy used during the course and post-course. Other studies have also mentioned mindful eating as a commonly used practice (Eyles et al. 2015), which was a frequently mentioned strategy from this study’s participants. Additionally, participants described increased levels of awareness, including greater body awareness and recognizing their limitations and subsequently considering ways to take care of their bodies (e.g., more rest and more activity). In other studies, increased awareness was linked to participants’ increased acceptance of themselves and their bodies, as well as decreased physical and psychological suffering (Luiggi-Hernandez et al. 2018; Penlington 2019; Tate et al. 2018; Visser et al. 2015). Furthermore, the strategies learned and implemented during the course were considered useful tools for managing their pain, such as breathing and focusing on external stimuli in their surroundings, rather than focusing on their pain. As one participant explained, “…I think it does divert your attention away from pain, and allow you to use other dimensions, and experience things in such a way that the pain doesn’t go away, but you’re not focusing on it. You’re focusing on something else.” Some participants noticed pain reduction, while other participants implemented the tools despite still feeling their pain and not expecting or trying to make it go away, such as one participant explaining “I’m still in pain, but at least I can use some of the tools that she [the teacher] gave me.”

The predominant barrier to practicing the meditations and mindful activities during the course and post-course was time. Participants were balancing multiple priorities and obligations, such as caretaking for family members or medical appointments, and making time to practice mindfulness was not compatible with their schedules and other responsibilities. External factors, such as the weather, also made some of the home practices challenging (e.g., mindful awareness while walking). These barriers align with previous studies noting time commitment and physical limitations as challenges in both the in-person and online space (Kinner et al. 2018; Tate et al. 2018). These results demonstrate a need for facilitators and the delivery of MBPs to consider participant demands and needs, such as considering shorter duration practices and adapting the activities in line with the participants’ physical needs, such as incorporating chair yoga rather than mindful walking for patients experiencing neuropathy. Likewise, these results may be connected to the most used mindfulness skills, such as mindful breathing (e.g., noticing one breath), noticing their surroundings (external awareness), and mindful eating. Integrating the practice of mindfulness into the participants’ everyday activities may be a way to address the time commitment and presenting the opportunity to practice “in the moment”, rather than adding the practices as a separate activity and commitment.

### Experiences in a RCT

Maintaining a sense of connection and continuity post-course was important to the participants, particularly due to the sense of connection and resonance that had been developed between class members and teacher. Although the teachers spent time during the final session (i.e., class eight) discussing ways to continue practice, including community classes and workshops, some participants felt abandoned after finishing the 8-week course. This is important for facilitators to consider, particularly in whether 8-week online courses could be longer in duration and whether offering ongoing forms of support and maintenance sessions for the participants post-course is warranted. This finding also highlights the importance of considering appropriate off-boarding practices so that abandonment and a sense of returning to feeling isolated are minimized for the participants. Previous research also describes participants wanting follow-up meetings (Berk et al. 2018), weekly and monthly drop-in, and refresher sessions (Kinner et al. 2018; Mackenzie et al. 2007). However, participants in these previous studies emphasized continuity in relation to maintaining an ongoing practice, while participants from this study also emphasized continuity of connection with their class members emphasizing the importance and potential value of online group classes. Additionally, technological challenges were noted, such as sound and Internet connection issues. Other studies have acknowledged the challenges of delivering Internet-based treatments (Kinner et al. 2018; Stjernswärd and Hansson 2017) and these are important considerations for future online MBP delivery platforms.

### Limitations and Future Research

Limitations for this study include collecting data from participants who were willing to participate in the interviews and share their experiences. Therefore, this study may be biased towards participants who benefited from the course. Additionally, the interviews were conducted between 19 and 293 days (mean = 100 days, median = 67) since course completion, which may have affected the accuracy of the data due to recall issues. On the other hand, participants who had more time to reflect on and integrate their experience may have had different insights or discoveries (i.e., longer time to reflect post-course). Furthermore, the interviewer (EK) had met the majority of the participants in-person due to the larger RCT study procedures, which may have impacted what the participants were willing to share, particularly in regard to their experiences in the research study**.**

Future research warrants further exploration of the benefits of online group MBPs such as learning relaxation techniques, pain and stress management, social support and effects of meaningful connections made with other participants and the teacher, and their underlying mechanisms and processes. Future research also needs to pay attention to adverse events such as any increased distress associated with the practice. Technological issues are worthy of consideration when developing and implementing future online MBPs, as well as preparing participants for potential technological challenges. Future MBPs and research studies should continue including the half-day session and practices that are convenient and easy to integrate in the participants’ daily lives during and post-course. Additionally, to address the lack of continuity post-course of study, supportive off-boarding practices, such as follow-up meetings or community groups, should be implemented and further tested.
